# The Wealth State Awareness Effect on Attention Allocation in People From Impoverished and Affluent Groups

**DOI:** 10.3389/fpsyg.2020.566375

**Published:** 2020-11-12

**Authors:** Shanshan Wang, Dong Yang

**Affiliations:** ^1^Department of Psychology, Southwest University, Chongqing, China; ^2^MOE (Ministry of Education) Key Laboratory of Cognition and Personality, Southwest University, Chongqing, China

**Keywords:** poverty, wealth state awareness, attention resource allocation, different income level, eye-movement study

## Abstract

Previous studies have shown that poverty influences cognitive abilities and that those who have a negative living environment exhibit worse cognitive performance. In addition, eye measures vary following the manipulation of cognitive processing. We examined the distinctive changes in impoverished and affluent persons during tasks that require a high level of concentration using eye-tracking measures. Based on the poverty effect in impoverished people, this study explored how wealth state awareness (WSA) influences them. It was found that the pupillary state indexes of the impoverished participants significantly changed when their WSA was regarding poverty. The results suggest that awareness of poverty may cause impoverished individuals to engage in tasks with more attention allocation and more concentration in the more difficult tasks but that a WSA regarding wealth does not have such effect on them. WSA has no significant effects on their more affluent peers. The findings of this study can contribute to research on WSA effects on impoverished individuals from the perspective of eye measures.

## Introduction

Poverty poses a major issue as it can restrict the development of human beings and society. Defined as a scarcity of financial resources or material possessions (Flythe, 2013; Akfirat et al., 2016), poverty has a cumulative long-term impact on cognition from childhood. It can hinder brain development (Cowell, 2008) and eventually reduce adult cognitive capacity (Evans and Schamberg, 2009), especially damaging attention (Hunt, 2011). However, studies of Mani et al. (2013) have shown that the damage caused by poverty is not irreversible. They found that cognitive performance improved after the farmers’ harvest (when they were rich) compared to before the harvest (when they were poor). This demonstrates that a change in the financial situation can affect the cognitive performance of poor people. Based on poverty damage cognition being not irreversible, we aim to explore the influence of poverty on people from the eye movement perspective, which suggests a close relationship between eye movement and cognition.

Mani et al. (2013) put forward the idea of the “scarcity mindset”; this posits that attentional resources are allocated to scarce things, while other important matters are ignored. Financial scarcity is one of the key factors that cause the scarcity mindset, and it is the most direct manifestation of poverty. Previous studies have found that the concept of money can have a dual effect on human behavior (Vohs et al., 2006; Zhou et al., 2009). For instance, Vohs et al. (2008) found that even subtle reminders of money could elicit some behavior changes, such as being less helpful, preferring solitary activities, and being less physically intimate, but also working harder. In short, the concept of money leads to alterations in human beings (Vohs et al., 2008). It is important to note that the scarcity mindset has a great effect on poor people and that a change in their financial state could change their cognitive performance. Thus, we hypothesized that wealth-related information may perhaps cause a certain mindset and may have distinct effects on people from families with different income levels. To explore it, we introduced a kind of awareness – the wealth state awareness (WSA) – which could be caused by wealth-related information.

As evidenced by previous studies mentioned above, awareness, including WSA, can have a profound influence on people. However, WSA has a more profound effect on poor people (Gopinath and Nair, 2015). Haushofer and Fehr (2014) found that a negative income shock had a greater negative effect on poor people than on rich people. In other words, income shock has different influences on impoverished and affluent persons. Thus, by combining the results of studies of the dual effect of money on human behavior and the influence of different wealth states on the cognitive capacities of poor individuals, we boldly propose the following hypotheses: changes in WSA might have different effects on the cognitive task performance of people who come from families with different income levels, reflecting on the attention resources allocation; and perhaps changes in WSA might have a more profound and different effect on impoverished people. These differences could be observed from the eye movements in this cognitive study, which can contribute to the understanding of the potential effect of WSA on people.

Due to the trait of the eye movement technique and negative effect of poverty on human’s attention, we conducted a research to explore people’s attention by eye movement methods, a method which can examine attention more directly. Attention has two basic characteristics: orientation and concentration. Orientation manifests the selection of stimuli. Concentration is shown as the inhibition of interference. As such, concentration requires a combination of attentional stability and resistance to distractions. The current study aims to explore the effects of WSA on participants’ attention from the perspective of concentration. Furthermore, eye movements and pupillary response are essentially motor movements in humans, which are closely associated with cognition (Schutz et al., 2011; Jang et al., 2014; Wang and Munoz, 2015). Not only are eyes used for scanning, but they also provide information on how and where the human gaze is based on intentions (Jang et al., 2014). It was shown that pupil size and variation were related to cognitive processing and visual information (Privitera et al., 2008; Wang and Munoz, 2015). This verifies the positive correlation between task-evoked pupil diameter, the cognitive load (Dionisio et al., 2001; Peysakhovich et al., 2015), and the attention required (Hoecks and Levelt 1993; Iqbal et al., 2004) to perform specific tasks. To illustrate, more fixations of eye movement are required to absorb more information from the surrounding environment (Gareze et al., 2008). It was also found that the length of fixations could reflect people’s attention (Hoecks and Levelt, 1993). The advantage of eye movement study is that it can directly provide insight into the spatial and temporal behaviors and mental effort in the tasks, reflected by fixation counts, fixation duration, pupil size, etc. And eye tracking is an objective method (Hessels et al., 2015). Basing on previous findings of the eye movement method and the aims of this study, we conducted visual searching studies by adopting the eye movement method. Eye-tracking metrics regarding fixations and pupil size were utilized to provide evidence for attentional allocation in this study.

Specifically, the design was to utilize the visual search task (VST) and a revised Stroop task, both of which require highly centralized attentional resources to process information and avoid interference. This study collected the data regarding only the physical parameters which are objective. In this study, we took into account the possibility that if it required participants to press the keys, there might be involuntary saccading, which perhaps would lead the view angle to be shifted or lead the tension to affect the pupil size. Therefore, behavioral performance or recording was not required in this study. Both tasks are based on the following ideas: the first task is used to test the basic attentional concentration, while the second task is designed to explore attentional concentration and inhibitory abilities with a higher difficulty level. To test inhibitory abilities, changing stimuli was employed in the VST as the distractor, and VST was used as a supplementary explanation for the revised Stroop task. WSA was created by manipulating the experimental design and instructions. All of the details are provided in the next section.

## Materials and Methods

### Sample Size Considerations

Sample size was determined *a priori* by utilizing G*Power 3.1.9.4 (Faul et al., 2007) for *F* tests (analysis of variance or ANOVA: repeated measures, within-between interaction). As for the action simulation paradigms (for reviews, see Horchak et al., 2014), we expected the large effect size to set the parameters as follows: effect size *f* = 0.25, alpha level = 0.05, and power = 0.95. The calculation suggested a minimum total sample size of 36 (repeated-measures ANOVA for group × condition × stimulus type × part × task type, in which the WSA condition and group were between-subject factors and others were within-subject factors). As we analyzed the different effects on each group, we computed the sample size as well with the same parameters, and the calculation suggested a minimum total sample size of 24 (repeated-measures ANOVA for WSA condition × stimulus type × part × task type, in which the WSA condition was a between-subject factor and others were within-subject factors).

### Participants

Sixty-seven volunteers were recruited from Southwest University. There were 45 females and 22 males, with a mean age of 19.6 years (*M* = 19.597, *SE* = 1.349). Thirty-one participants were selected from poor families, and 36 were from more affluent families. All participants were native Chinese speakers who have normal or corrected-to-normal vision and are naïve to the purpose of the experiment. Based on the family socioeconomic status, the participants were divided into a poor group (PG) and a rich group (RG; those who were more affluent) by referring to the Income Standard of Poverty Households of Southwestern China. To be more rigorous, we also computed the per capita income by dividing the total income of the household by the square root of the household size (Smeeding et al., 1988). We then defined the PG and RG using the standards of poverty and calculated the median split of the per capita income to verify this. Finally, the PG included participants who had grown up in and lived in poor families, with a current per capita income of less than 1,300 RMB for urban residents and less than 1,000 RMB for rural residents. The RG included the remaining participants who had rarely experienced poverty in their childhood or until now and whose per capita income exceeded the standard. Table 1 showed the descriptive statistics for the per capita income of groups.

**Table 1:** Descriptive statistics for the per capita income of groups.

Group	Urban residents		Rural residents	
	Mean	SD	Mean	SD
PG	878.278	619.462	576.692	154.206
RG	1,849.346	244.972	1,420.000	300.051

This research was approved by the ethics committee of Southwest University and was performed in accordance with relevant guidelines. All volunteers provided their written informed consent before the experiment.

### Design

**Figure 1:**
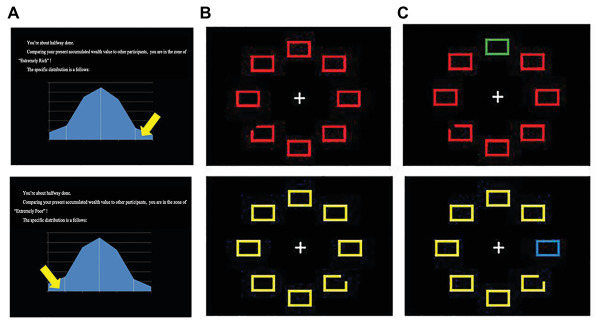
**(A)** Examples of the false feedback, **(B)** example stimuli without distractor of the visual search task, and **(C)** examples of the distractor in the present study.

A VST and a Chinese Stroop Search Task (CSST) were conducted on a computer. The CSST combines the original version of the Stroop task with a VST. Regarding WSA manipulation, the instruction notified participants that there would be a wealth value conversion and accumulation based on the performances in the tasks, which was accomplished by computer back-office automatic processing. In each trial, the less reaction time spent to find and focus on the target stimuli and the more stable to focus on the target stimuli (that is, the more successfully ignore distractions), the higher the converted wealth value. The accumulated wealth value of each trial would range from 0.1 to 1. After the end of the experiment, the accumulative wealth value would be compared with the wealth value of others collected in this study, and a distribution report of wealth value will be issued to the participants. Fake feedback was then used to prime WSA by randomly showing the participants that they were extremely poor (EP; “you are located in the extremely poor section”) or extremely rich (ER; “you are located in the extremely rich section”; see Figure 1A). After receiving the feedback, the participants were required to perform the same tasks once more. Therefore, the experiment was divided into two parts based on the feedback: Part 1 and Part 2.

The experiment was a 2 (group: PG vs. RG) × 2 (WSA: EP vs. ER) × 2 (part: 1 vs. 2) mixed factorial design for the two tasks (VST or CSST) with three stimulus types (pre-distractor vs. distractor vs. post-distractor), in which the group and WSA were between-subject variables and the remaining variables (part, tasks, and stimulus types) were within-subject variables. Due to the different display times of each kind of stimulus and the task requirements, we did not analyze the eye movement parameters regarding duration in the current study. Therefore, measurements of eye movement on the first run fixation count percent (FRFCP), fixation count, and pupil size were conducted. Fixation count is defined as the number of fixations within the area of interest (AOI; Lin and Lin, 2014), and FRFCP is the total number of fixations on AOI when entered for the first time of all number of fixations in the trial (Glaholt and Reingold, 2011). It is deemed that the number of fixations in a search task can reflect task difficulty (Vlaskamp and Hooge, 2006; Köerner and Gilchrist, 2008). Furthermore, researchers confirmed that pupil size variation could reflect the human’s emotion, arousal, stress, cognitive load, or efforts during tasks (Goldwater, 1972; Beatty, 1982; Hoecks and Levelt, 1993). Furthermore, because WSA was a between-subject factor in this study, we calculated the mean pupil size of the fixation point of each participant to be the individual baseline. Then, the individual baseline was subtracted from the pupil size of each individual’s response in the tasks. Therefore, the change of pupil size was finally analyzed in the current study.

The statistical analysis was a mixed model of 2 (group: PG vs. RG) × 2 (WSA: EP vs. ER) × 2 (part: 1 vs. 2) × 2 (task: VST vs. CSST) × 2 (distractor: pre-distractor vs. distractor) repeated-measures ANOVA. The significance level (*p* < 0.05) was adjusted according to sphericity violations, and the Greenhouse-Geisser correction was employed. When significant interactions emerged, the data were analyzed using the Bonferroni correction by *post hoc* analysis for simple effects. All analyses were performed using the Statistical Package for Social Sciences (SPSS version 22.0; IBM Corp., Armonk, NY, USA).

### Materials and Procedure

#### Visual Search Task

The visual search array was composed of eight geometric shapes that were arranged around a central fixation point; that is, seven rectangles and one rectangle that was randomly missing a corner (Figure 1B). Their height-to-width ratio was above 17:12, and the outlines were 0.4 cm thick. The rectangle with the missing corner was the target, the remaining seven shapes were uniformly colored and shaped non-targets (neither target nor distractor), and one colored rectangle was used as a distractor (Figure 1C). The distractor’s color was varied using the complementary color of the primary colors. Green, red, yellow, blue, and white were utilized in this study. The target and distractor were randomly presented in one of these colors.

**Figure 2:**
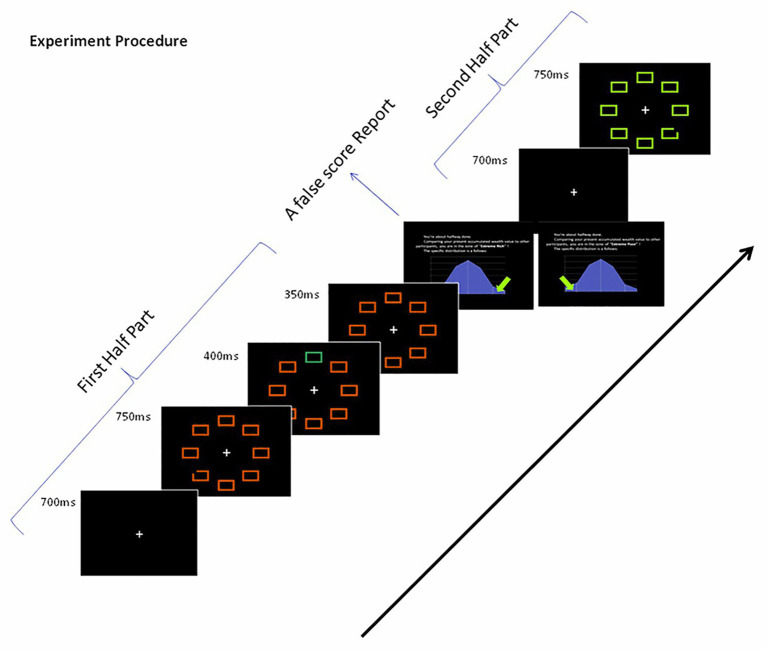
Sample of the visual search task trial sequence.

Each trial began with a white fixation point presented for 700 ms in the center of a black screen. All rectangles appeared in fixed positions for 750 ms. After this, one of the non-targets changed color for 400 ms as the distractor. Then, it changed back to the original color and was presented for another 350 ms (see Figure 2). Both types of fake feedback were randomly shown to the participants (see Figure 2) during a 5-min break. Before beginning the experiment, the participants were instructed to quickly find the shape that was different from the other shapes, to maintain fixation on it, and to ignore the changing distractor. A total of 180 trials were conducted, which consisted of two random blocks of 90 trials each. Five practice trials were completed before the formal experiment began for each part.

#### Chinese Stroop Search Task

**Figure 3:**
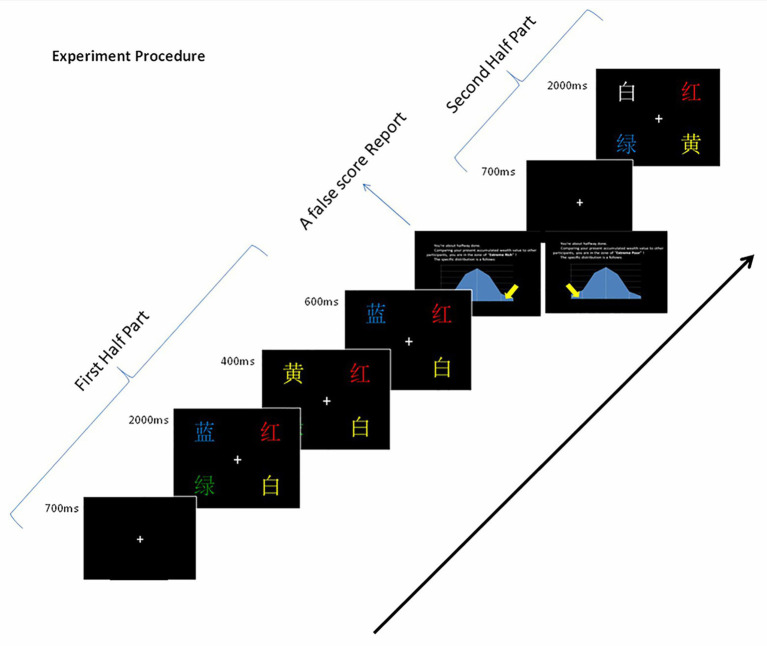
Sample of the Stroop task trial sequence.

Five color words (red, green, yellow, blue, and white) were drafted in Chinese. Four words randomly appeared in each display; three words were congruent in terms of their color and meaning, and one incongruent word was used as the target. The remaining congruent word was then quickly replaced with one of the three congruent ones as the distractor. Each trial began with a white fixation point that was displayed for 700 ms in the center of the black screen. The stimuli appeared in fixed positions for 2,000 ms. After this, a congruent word randomly replaced one of the non-targets (congruent word) for 400 ms as the distractor. For example, four words were displayed in a certain trial, such as red in white ink (target stimulus), green in green ink, yellow in yellow ink, and white in white ink (non-target stimuli); and a distractor, blue in blue ink, replaced yellow. The distractor then disappeared, and all of the original stimuli were presented for another 600 ms (see Figure 3). The instructions and procedure were the same as in the VST. A total of 210 trials were conducted, which were mixed across three random blocks that had 70 trials each. There were five practice trials at the beginning.

### Apparatus

An SR Research EyeLink 1000 eye tracker (SR Research, Mississauga, ON, Canada) was used to record the participants’ eye movements with a sample rate of 1,000 Hz. A cathode ray tube (CRT) monitor is connected to a Pentium IV 3.2-GHz mainframe. All stimuli appeared on the 21-in. CRT monitor with an 85-Hz refresh rate, a 0.1° spatial resolution, and a 1,024 × 768-pixel resolution, and they were viewed at the fixed distance of approximately 50 cm. Participants were positioned using a chin rest. Before the formal task began, the experimenters inspected the eye-tracking trajectory using the EyeLink proprietary algorithm. After the fixation was measured, nine points were presented randomly on the default positions using the calibration techniques. The calibration was validated by repeatedly measuring the pupil detection and corneal reflection. “Good” or “perfect” reports were accepted as accurate calibration. Following this, the drift correction was also performed. If the drift was greater than 5°, it was recalibrated. Furthermore, the pupil size unit used in this study is pixels. When the participants blink, the eye tracker fails to detect the eyes, the pupil, or the corneal reflection, resulting in loss of data in the recording. Therefore, the lost data were removed, and the means were calculated for analysis.

## Results

The experiment revealed significant interaction effects for task × distractor × group [*F*(1, 63) = 5.101, *p* = 0.027, *ŋ*_p_^2^ = 0.075] and task × distractor × WSA [*F*(1, 63) = 5.521, *p* = 0.022, *ŋ*_p_^2^ = 0.081] of the change in pupil size. The *post hoc* analysis for the simple effects of interaction effects did not find significant differences of group or WSA.

**Figure 4:**
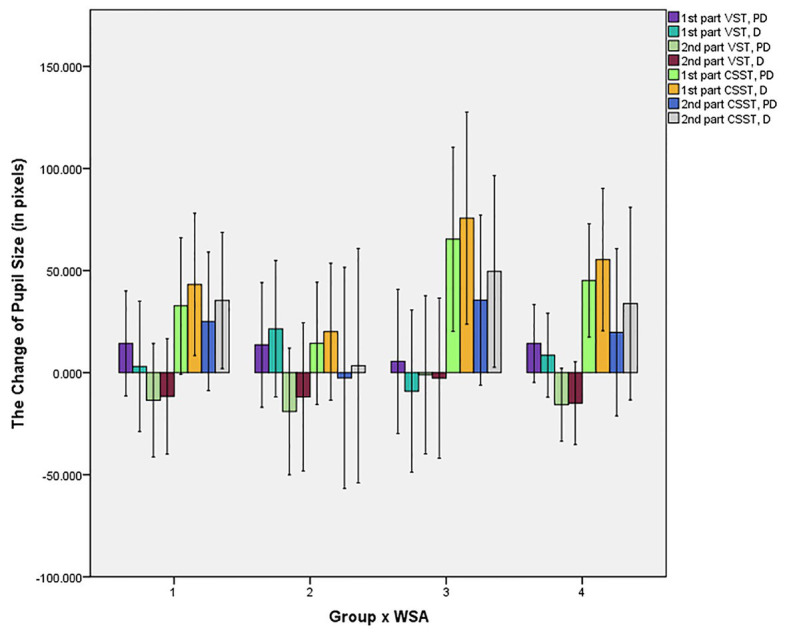
Bar chart of differences of the change in pupil size (in pixels) in all conditions. PD, pre-distractor; D, distractor; Condition 1, PG with EP feedback; Condition 2, PG with ER feedback; Condition 3, RG with EP feedback; Condition 4, RG with ER feedback.

Furthermore, we found several interesting differences in both groups by further analysis; that is, the WSA effect resulted in significant differences in PG as compared to the RG, which did not exhibit significant differences. Repeated-measures ANOVA for task × part × distractor × WSA was conducted, in the PG or in the RG separately. Specifically, regarding the change in pupil size, it was found that there was a significant interaction effect for task × distractor × WSA [*F*(1, 29) = 9.550, *p* = 0.004, *ŋ*_p_^2^ = 0.248] in PG. The *post hoc* analysis for the simple effects found that, with the EP feedback, the change in pupil size of CSST was significantly larger than that of VST whether it was for the pre-distractor or the distractor [pre-distractor: *F*(1, 29) = 5.763, *p* = 0.023, *ŋ*_p_^2^ = 0.166; distractor: *F*(1, 29) = 11.524, *p* = 0.002, *ŋ*_p_^2^ = 0.284]. The change in pupil size of the distractor was significantly larger than that of the pre-distractor in CSST [*F*(1, 29) = 6.524, *p* = 0.016, *ŋ*_p_^2^ = 0.184]. We did not find any significant difference when ER feedback was shown to them (see Figure 4). Repeated-measures ANOVA for task × part × distractor × group was conducted, in the condition of EP and ER separately. It did not find any significant differences regarding group.

Regarding the pupil size, the difference of pupil size for the fixation point was analyzed as well, by conducting *t*-test for group × mean pupil size of the fixation point. It did not reveal significant differences between both groups (*t* = −0.728, *p* = 0.469; PG: M ± SD = 572.814 ± 176.875, RG: M ± SD = 603.090 ± 163.400).

**Figure 5:**
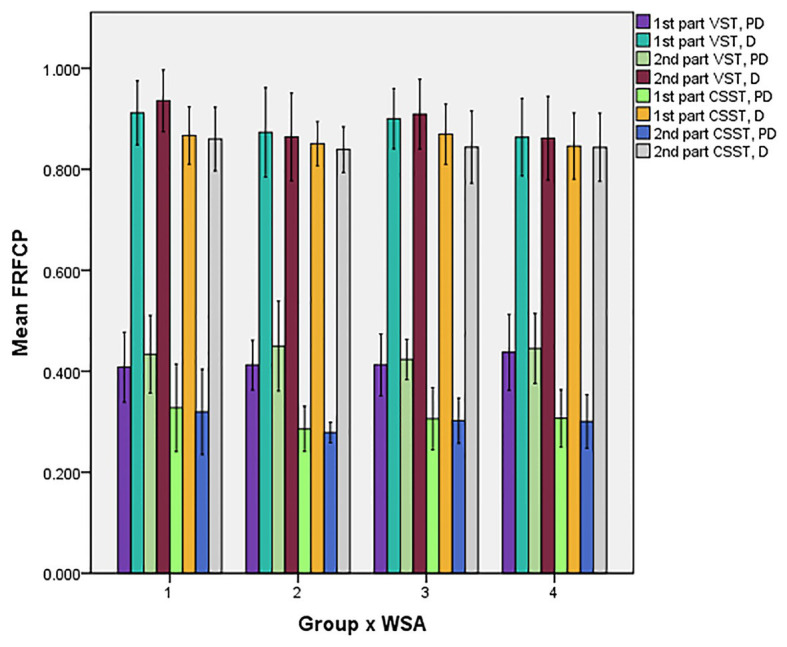
Bar chart of differences of FRFCP in all conditions. PD, pre-distractor; D, distractor; Condition 1, PG with EP feedback; Condition 2, PG with ER feedback; Condition 3, RG with EP feedback; Condition 4, RG with ER feedback.

A significant interaction effect was also found for task × distractor × WSA of the FRFCP [*F*(1, 29) = 5.742, *p* = 0.023, *ŋ*_p_^2^ = 0.165] in the PG. The *post hoc* analysis found that the FRFCP of CSST was significantly higher than that of VST in all conditions (*p*s ≤ 0.001), except for the distractor with ER feedback; all FRFCPs of the distractor were significantly higher than that of the pre-distractor (all *p*s < 0.001, see Figure 5).

In other words, there were no significant interaction effects of pupil size change [*F*(1, 34) = 0.576, *p* = 0.453, *ŋ*_p_^2^ = 0.017] or of FRFCP [*F*(1, 34) = 0.209, *p* = 0.651, *ŋ*_p_^2^ = 0.006] of task × distractor × WSA or other significant differences regarding WSA in RG.

## Discussion

The present study utilized eye-tracking methods to investigate the WSA effect on resistance to interference in people from families with different income levels by combining revised visual searching tasks with a distractor. The results revealed that eye measures did not have typical interaction effects on the group and WSA. Based on the pupil size for fixation of the RG individuals, it showed slightly larger pupil size than that of the PG individuals, although the differences were not significant. We think that perhaps due to this difference of the change in pupil size between both crowds or perhaps because RG individuals may devote more mental resources to the tasks than PG peers, thus, the increase and decrease of changes might not be directly reflected in the significant interaction effect in the tasks. The results indicated that WSA had more significant effects on the impoverished group than on its counterpart. Generally, we found that the WSA of EP had broader effects on the impoverished group than had the WSA of ER, shown by the eye measures of the pre-distractor and distractor.

Although image processing and word processing are based on different neural mechanisms, the studies were utilized to explore concentration in participants to require them to only sustain attention on the obvious physical property and resistance to interference. Therefore, VST and CSST are the same based on the final reaction requirements. It is a probability that WSA will influence people’s cognitive psychology, especially when they are aware of the poverty. This study found that WSA had a wider range of effects on impoverished persons instead of their more affluent peers, as shown by eye measures. With this, we argue that there may be distinct effects of WSA in people from families with different income levels on abilities of concentration and that it could be reflected by the eye movement of individuals. However, we did not find any significant effects of WSA on affluent people, and WSA had more significant effects in impoverished ones.

When impoverished people’s feedback was regarding EP WSA, it was found that the change of their pupil became larger in size, and they had less FRFCP of CSST for stimuli than that of VST. The feedback about poverty resulted in more significant task differences in the distractor set as shown by the FRFCP and can lead to significant differences between pre-distractor and distractor situations in CSST in the impoverished group, as shown by the change in their pupil size.

From these findings, we can surmise that feedback regarding their performance would have no significant effects on affluent people’s concentration in the cognitive performance but would have a modulation effect on impoverished people. Impoverished people will make some change to their performances in the following tasks, which perhaps can improve their task goals. However, because the data of behavioral responses were not collected, we could not directly posit that WSA changed behavior; however, the collected data of eye movement did have significant differences, and it has been established in previous studies that eye movement is closely related to cognition.

The findings show that WSA perhaps has a deeper effect on impoverished people, which resulted in more attention allocation in the tasks performed and showing significant differences on eye movement. In particular, when the feedback about EP was shown to the impoverished group, their pupil size grew larger, and there was less FRFCP of CSST than that of VST. Previous studies have shown that fixations could indicate how people acquire information, and the number of fixations in the search task could reflect task difficulty (Vlaskamp and Hooge, 2006; Köerner and Gilchrist, 2008; Alotaibi et al., 2017). Due to this study requiring participants to find and focus on the targets, less FRFCP means more concentration on the control target (that is, pre-distractor) and stronger anti-interference capability (to distractor). The findings that there was a lower number of fixation percentage in the first run suggest that impoverished people concentrate more and maintain stronger ability to avoid interference in the more difficult tasks compared to the easier tasks when they are aroused with the awareness of poverty. The pupil size differences help prove this point. In addition, pupil size variation also reflected the transient variations of a subject’s effort of the performance (Goldwater, 1972). Thus, the pupillary response could be considered as a potential measurable trait to help recognize people’s implicit intentions and behaviors (Jang et al., 2014).

The findings regarding pupillary variation suggest that, when the poverty awareness was aroused in impoverished people, the pupil size of CSST was significantly larger than of VST and that the pupil size of the distractor was significantly larger than the pre-distractor (control stimuli), especially in the CSST. Therefore, it is suggested that poverty awareness has a deeper influence on impoverished people and that the differences may become more apparent as the task difficulty increases. However, this needs to be examined further. Combined with the differences of FRFCP, it was shown that impoverished people were more sensitive to the awareness of poverty, and it could elicit some changes in cognitive performances. In contrast, the awareness of wealth has no such influence on the impoverished people, and WSA has no obvious effects on the more affluent subjects’ cognitive performances as well.

Mani et al. (2013) verified that rich and poor people treat tasks differently – that is, poor people are usually more engaged in the task and pay more attention. Thus, it is posited that poor people apply different processing strategies from those applied by rich people. Hence, the findings in this study may help to highlight these points, as awareness of poverty can lead impoverished people to have a higher involvement in the tasks. It is suggested that WSA of EP might play an active role in the impoverished individuals’ cognitive ability, which showed that more attentional resources are utilized. Mani et al. (2013) posited that poverty affects cognitive resources, and Bertrand et al. (2004) also suggested that internal factors promoted development in poor people.

Therefore, the findings of this study suggest that impoverished people can show an increased and outstanding cognitive ability within the WSA of poverty with flexible processing strategy such as utilizing more mental resources and making more effort. Furthermore, it has previously been found that poverty is a circumstance in which individuals have deficits in cognitive function (Nelson et al., 2007; Evans and Schamberg, 2009; Forssman et al., 2017). The findings of this study reaffirm the differences in cognitive capacity about attention allocation and concentration abilities between impoverished and more affluent people.

Generally, the aforementioned eye measure findings suggest that awareness of poverty does have a promoting influence on impoverished persons to allocate more cognitive resources on more difficult tasks. However, WSA has no significant effects on their more affluent peers.

It is important to note that there are limitations in this study. First, due to the pursuit of a more authentic response of the pupil in individuals, we did not collect behavioral data. If behavioral data were collected, it would perhaps find other differences in behavioral performances. Second, because we aimed to analyze the results of the corresponding response before and after the feedback only, the presentation times of stimuli in both tasks were not the same. If we designed them to be presented with the same length of time, we could have several other interesting findings by analyzing them together. However, it is unlikely that it will yield any differences due to either the ceiling effect or the floor effect. To address these limitations, we will explore them by conducting further studies.

## Data Availability Statement

The datasets presented in this study can be found in online repositories. The names of the repository/repositories and accession number(s) can be found in the article/supplementary material.

## Ethics Statement

The studies involving human participants were reviewed and approved by the ethics committee of Southwest University. The patients/participants provided their written informed consent to participate in this study.

## Author Contributions

SW and DY contributed to the conception and design of the study and wrote the first draft of the manuscript. SW organized the database and performed the statistical analysis. Both the authors contributed to manuscript revision, read and approved the submitted version, and agreed to be accountable for the content of the work.

### Conflict of Interest

The authors declare that the research was conducted in the absence of any commercial or financial relationships that could be construed as a potential conflict of interest.
